# American Crow Brain Activity in Response to Conspecific Vocalizations Changes When Food Is Present

**DOI:** 10.3389/fphys.2021.766345

**Published:** 2021-11-18

**Authors:** LomaJohn T. Pendergraft, John M. Marzluff, Donna J. Cross, Toru Shimizu, Christopher N. Templeton

**Affiliations:** ^1^School of Environmental and Forest Sciences, University of Washington, Seattle, WA, United States; ^2^Department of Radiology and Imaging Sciences, University of Utah, Salt Lake City, UT, United States; ^3^Department of Psychology, College of Arts and Sciences, University of South Florida, Tampa, FL, United States; ^4^Department of Biology, Pacific University Oregon, Forest Grove, OR, United States

**Keywords:** American crow, 18F-fluorodeoxyglucose PET imaging, social stimuli, brain activity, nucleus taenia of the amygdala (TnA), caudal nidopallium, vocalizations, multimodal stimulus

## Abstract

Social interaction among animals can occur under many contexts, such as during foraging. Our knowledge of the regions within an avian brain associated with social interaction is limited to the regions activated by a single context or sensory modality. We used 18F-fluorodeoxyglucose positron emission tomography (FDG-PET) to examine American crow (*Corvus brachyrhynchos*) brain activity in response to conditions associated with communal feeding. Using a paired approach, we exposed crows to either a visual stimulus (the sight of food), an audio stimulus (the sound of conspecifics vocalizing while foraging) or both audio/visual stimuli presented simultaneously and compared to their brain activity in response to a control stimulus (an empty stage). We found two regions, the nucleus taenia of the amygdala (TnA) and a medial portion of the caudal nidopallium, that showed increased activity in response to the multimodal combination of stimuli but not in response to either stimulus when presented unimodally. We also found significantly increased activity in the lateral septum and medially within the nidopallium in response to both the audio-only and the combined audio/visual stimuli. We did not find any differences in activation in response to the visual stimulus by itself. We discuss how these regions may be involved in the processing of multimodal stimuli in the context of social interaction.

## Introduction

Social animals must filter, process, and act upon a variety of information when they assemble and interact with one another; they send and receive signals across multiple sensory modalities, observe interactions between conspecifics, and evaluate the intentions of others toward themselves, all the while remaining vigilant for danger and attempting to maximize their access to any resources in the area. This cognitive demand requires a brain with a high degree of processing power (Dunbar, 1998, 2009). Most species known to possess such a brain are mammals (such as primates or cetaceans) or birds (such as corvids or parrots). Despite convergently evolving advanced cognitive capabilities, these two classes diverged approximately 300 million years ago (Burt et al., 1999), resulting in numerous structural differences between mammalian and avian brains (Shimizu, 2001).

Aside from regions and systems homologous to both clades, our understanding of the inner workings of the avian brain is limited relative to mammals, although a surge of research conducted over recent decades has made considerable progress in filling this gap (Reiner et al., 2004; Jarvis et al., 2005; Wada et al., 2017; Ksepka et al., 2020). Much of this work has focused on determining the functions of, and connectivity between, individual brain regions (see Cowan et al., 1961; Karten et al., 1973; Nottebohm et al., 1976; Wild et al., 1993; Shanahan et al., 2013 as examples). Fewer studies have examined how systems within the avian brain function holistically, though our extensive understanding of the avian song control system (Konishi, 1985; Brenowitz and Beecher, 2005; Brainard and Doupe, 2013) remains a notable exception. While scientists have uncovered numerous brain regions associated with the avian social network, such as the lateral septum, nucleus taenia of the amygdala (TnA), anterior hypothalamus, ventromedial hypothalamus, preoptic area, and potentially the dorsal arcopallium (Cooper and Erickson, 1976; Goodson, 2005; Atoji et al., 2006; Nishizawa et al., 2011; Ondrasek et al., 2018), how these regions interact with other areas of the brain under different social situations remains unclear. For example, counter-singing between neighboring rivals, fights over access to resources, courting a potential mate, and recruiting to food are all examples of social behavior, yet likely involve different regions/systems within the brain due to varying social contexts and sensory modalities. Scientists must also consider how the brain integrates multimodal sensory information, as animals regularly communicate social information using more than one modality (Horn, 1983; Gopher et al., 1996) and usually pay more attention to multimodal signals, regardless of whether each modality is transmitting redundant or non-redundant information (Partan and Marler, 1999).

American crows (*Corvus brachyrhynchos*) are songbirds noted for their intelligence and complex social dynamics; they guard territories and regularly fight among themselves (occasionally escalating to the death of one of the belligerents), yet they also cooperate to mob predators and roost communally in large numbers (Marzluff and Angell, 2005). Much of this complex social interaction can be observed when crows congregate around an ephemeral food source; while they certainly spend time obtaining food, they also use these occasions as opportunities to gauge their position within the local dominance hierarchy, search for prospective mates, and learn about potential rivals (Kilham, 1990; Marzluff and Angell, 2005, 2013). Crows exchange much information via vocalization; as a result, such gatherings can become quite noisy as crows communicate with one another (Pendergraft and Marzluff, 2019).

To supplement insights from behavioral observation, Positron Emission Tomography combined with the radiotracer 18F-fluorodeoxyglucose (FDG-PET) can be used to better understand the brain activity of animals from various stimulus conditions. In this brain imaging modality, the FDG, a glucose analog, is injected into the body and distributes systemically. The uptake of FDG within the brain is preferential to regions of increased activity; therefore, the levels of radioactivity in a brain region (as measured by PET) act as a surrogate marker of brain activity (Jonides et al., 1993). An advantage of FDG-PET over other *in vivo* imaging modalities is that the subject can be awake (unanesthetized) and free from restraints or attached apparatus (which can cause stress to an unhabituated animal, confounding the results) during the stimulation period, as the circulating FDG is trapped in the tissue of the active brain region but cannot be further metabolized by glucose-6-phosphotase within the glycolytic pathway (Newberg et al., 2002). The subsequent PET imaging can be performed under anesthesia to assess the brain activity during the prior stimulation period (Marzluff et al., 2012).

This methodology has been used for various studies to identify distinct regions within the crow’s brain that respond to specific stimuli (Marzluff et al., 2012; Cross et al., 2013; Swift et al., 2020). For example, wild crows respond to a variety of dangerous stimuli by giving alarm calls and mobbing the threat, yet an innate fear (a red-tailed hawk, *Buteo jamaicensis*) caused increased activity in the caudal nidopallium, whereas a learned fear (human who was previously antagonistic) activated the amygdala and a novel fear (unfamiliar human holding a dead crow) stimulated the hippocampus (Cross et al., 2013). While other methods can reveal activity at the regional/neuronal level or the connectivity between regions, such as implanting microelectrodes (Kita and Wightman, 2008) or antegrade/retrograde degeneration (McGeer and McGeer, 1980), respectively, PET imaging allows one to examine brain activity holistically (albeit indirectly via FDG uptake) and make inferences about regional connectivity based on the active regions.

Here, we conducted a 18F-fluorodeoxyglucose PET imaging study with the objective of determining how the American crow brain holistically functions in response to different sensory modalities associated with communal feeding events, with the secondary objective of understanding how the avian brain processes multimodal sensory information. We compared the baseline brain activity (as measured by the relative FDG activity) in wild crows during a control condition (viewing an empty stage) to their brain activity when hearing conspecifics foraging, seeing a preferred food item, or both hearing foraging and seeing food. We selected four regions *a priori* that we hypothesized would be activated in response to specific conditions. Because vocalizations encode social information, we expected the audio stimulus to cause an increase in activity in regions of the brain associated with social interaction, such as the (1) amygdala (specifically TnA) or the (2) lateral septum; multiple studies point to these regions as being involved in the vertebrate social network (Cooper and Erickson, 1976; Goodson, 2005; Nishizawa et al., 2011). If the calls encode information about food (such as presence, quantity, or quality), we hypothesized that the (3) hypothalamus, which is involved with motivation and food regulation (Wright, 1968; Kuenzel, 1994; Kuenzel et al., 1999; Primeaux et al., 2013), would also increase in activity in addition to the regions associated with social interaction; for the same reasons, we predicted this region would also increase in activity in response to the sight of a preferred food item. Finally, because the (4) thalamus filters, organizes, and relays information gathered by the senses to other brain regions (Bentivoglio et al., 1993), we expected to see increased activity here in response to the combined multimodal stimuli (sound of conspecific vocalizations and sight of food item), as this region will be processing additional information.

## Materials and Methods

### Capturing and Housing Crows

We captured wild American crows near Woodinville, WA, United States, as they departed a large communal roost. We lured birds from flocks with bread, trapped them using a net launcher, and preferentially selected individuals that were likely adult males (determined by plumage color and wear, mouth color, and overall size, Emlen, 1936). We caught two groups of crows outside of the breeding season and held them for several months each (9 crows from October to December 2015 and 8 crows from January to March 2016) in a protected outdoor aviary at the University of Washington, Seattle. The crows were individually housed in adjacent cages (measuring 1.8 × 2.1 × 2.4 m) separated by wire mesh. We provided crows with a rotating diet of assorted meats, eggs, grain, fruit, and dried dog food *ad libitum*. After identifying the crows’ most preferred food item (half of a fried chicken patty) by observing which food item was preferentially consumed first, we began wrapping it in plastic food film prior to giving it to them to match the food presentation during the imaging process. Crows easily removed the plastic film prior to consuming the chicken, and they habituated to receiving their favorite food item in this presentation.

### Imaging the Crows

We imaged up to three crows per day, using a Siemens Inveon PET/CT system. The scanning process consisted of a 20 min microPET scan, followed by a CT scan in the docked and coregistered microCT scanner. The scanners share a multimodality bed and have a bore diameter of ∼12 cm. The PET field of view was approximately 8 × 13 cm^2^ while the CT field of view was 7.9 cm × 13.3 cm; both included the entire brain with a slice thickness of approximately 0.1 mm. The scanner bed contained a pressure pad, which we used to monitor the crow’s breathing (and thus depth of anesthesia) during the scan process.

The evening before a bird was scanned (typically 1,600–1,700), we removed it from its aviary cage, placed it in a sock to keep it docile, and carried it across campus to the imaging laboratory, where we placed it in a small wire cage (1 × 0.5 × 0.5 m, hereafter imaging cage) within a fume hood to acclimate overnight. The imaging cage contained water but not food, ensuring crows fasted for at least 14 h prior to imaging to control for variable blood glucose levels influencing FDG uptake. We covered the cage with a blanket to keep the bird calm; it could hear the ambient noise of the equipment but could not see into the imaging lab.

**FIGURE 1:**
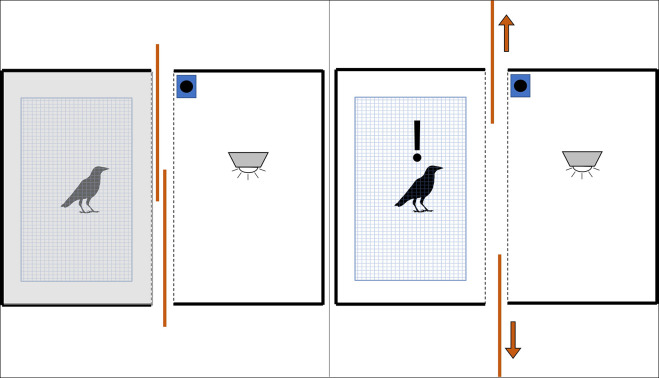
Graphic of experimental layout during stimulus phase (top-down view). The fume hood surrounding the crow’s cage blocked all view of the surrounding laboratory. When we closed the sliding panels between the crow’s cage and the stimulus stage (left), the crow was plunged into relative darkness. We revealed the interior of the well-lit stage (and any stimulus within it) by opening the sliding panels (right).

On the morning of the experiment, we removed the acclimated crow from its cage, covered the bird’s head with a cloth to calm it, and gave it an interperitoneal injection of approximately 1 mCi of [^18^F] Flourodeoxyglucose (FDG) (exact volume adjusted to account for radioactive decay and the bird’s weight, ranging from 0.05 to 0.10 mL). After injection, we returned the crow to the covered imaging cage. During the next 3 min, we positioned our stimulus stage (see section “Experimental Stimuli” below) in front of the covered cage and removed the blanket covering the wire cage- the crow remained in relative darkness because the fume hood blocked the view to the lab while the stimulus stage’s closed sliding panels prevented the crow from seeing the illuminated stage interior (Figure 1). Three minutes post-injection, we opened the sliding panels to reveal the stimulus (see section “Experimental Stimuli” below). For the following 10 min (hereafter referred to as the “stimulus phase,” see Supplementary Table 1), we used the sliding doors to alternatively reveal the stimulus to the crow for 60 s, then hide it for 30 s (seven exposures and six associated breaks total). After the stimulus phase ended at 13 min post-injection, we again removed the crow from the cage, covered its head with a cloth, and anesthetized it via a custom nose cone with 5% isoflurane in oxygen with a flow rate of 300–800 mL/min before placing it in the scanner (we reduced isoflurane concentration to 2.5–3% after the crow was fully induced). We used Velcro straps to secure the anesthetized crow to the scanner bed before starting the imaging process 26 min post-injection. After the scan was complete, we secured the crow in hand until it fully recovered from anesthesia (indicated when it regained the ability to grip with both feet), before returning it to the cage. We kept the crows in the imaging lab for 20 h (the time required for ^18^F radioactivity to decay to acceptable levels), after which we returned them to the aviary.

We imaged all crows twice- the first scan for all birds was the control while the second scan introduced one of three different stimuli (see section “Experimental Stimuli” below). This ordering was to prevent carryover of the crow’s previous experience biasing the control scan (Swift et al., 2020). We waited at least 1 week between scans for most crows, although one crow received its second scan 4 days after the first due to logistical constraints.

### Experimental Stimuli

As part of the imaging process (see section “Imaging the Crows” above), we presented all experimental stimuli within a (1.2 × 0.6 × 0.45 m) wooden stage designed to block the crow’s view of the imaging laboratory/personnel (thereby removing potential confounding sources of distraction) and to standardize the background color, light intensity, and light angle between trials (Figure 1). The stimulus stage always contained an LED light on the ceiling and a Bem wireless HL2022A speaker placed far enough to the side to be considered out-of-view for the experimental crow. The front of the stimulus stage had two overlapping sliding panels, which we used to reveal or hide the stage interior- in addition to blocking the crow’s view of the stimulus, the panels also blocked nearly all the light from the internal LED, increasing the visual contrast between showing and hiding the stimulus. The panels opened from the center of the crow’s view of the stage so that the crow’s eyes received equal stimulation. We used this configuration as the control stimulus (*n* = 13).

During the crows’ second scan, we introduced either a visual stimulus (*n* = 4), auditory stimulus (*n* = 5), or combination of audio/visual stimulus (*n* = 4) to the stimulus stage interior. For the visual food-associated stimulus, we placed a fried chicken patty directly underneath the LED light in the center of the stage. We wrapped the food in plastic food film to reduce its scent profile, which matched how the crows received this item in the aviary. For the auditory food-associated stimulus, we used the internal wireless speaker to play a 60 s recording (see Supplementary Figure 1 for a sample audio stimulus spectrogram) of roughly 30–40 crows vocalizing as they foraged around a food source (a pile of bread), which was synchronized to begin when the stimulus stage interior was revealed (see section “Imaging the Crows” above and Supplementary Table 1) and end when it was hidden. We recorded 22 min of crows vocalizing at the capture site (1 day prior to capture) in WAV format using a Marantz PMD-671 solid-state recorder and a Sennheiser MKH 20-P48 microphone contained within a Telinga Universal Parabolic Dish MK2 housing. From the 22-min master track, we selected ten 60 s duration intervals which were relatively free of other noises, such as passing cars. We controlled stimulus amplitude by normalizing the peak amplitude using Audacity (Audacity Team, 2015) and keeping the source and speaker volume consistent between trials (mean = 73 dB, SD = 3 dB). We reduced other potential acoustic confounding factors by excluding recordings with crow alarm vocalizations and randomly assigning a unique exemplar to each crow that received either an auditory or combined stimulus. For the combined A/V stimuli, we simultaneously showed the wrapped fried chicken while playing the assigned audio file of conspecifics vocalizing during each reveal of the stimulus stage interior.

### Behavior During Imaging

To better gauge their level of attention toward the stimulus stage and to control for factors which may influence FDG uptake within the brain (Marzluff et al., 2012; Cross et al., 2013), we used a GoPro Hero 4 camera to record (30 fps) the gaze time, blink rate, and amount of movement of each crow during the stimulus phase of the imaging process.

Avian brains are highly lateralized (Rogers and Anson, 1979), so we measured the gaze time from each eye to verify that any observed differences in hemispherical activity were not due to the bird preferentially using one eye to view the stimulus over the other (Mench and Andrew, 1986). We tracked each eye’s gaze time independently from the other eye, e.g., we added gaze time to each eye if the bird binocularly gazed directly into the stage. We also used gaze time to measure a crow’s level of interest in the stimulus being presented, and thus only recorded gaze when the stimulus stage’s interior was revealed and visible to the crow (see section “Imaging the Crows” and Supplementary Table 1).

We measured blink rate to verify that the crows were not threatened by any of the presented stimuli or prior experience in the scanning apparatus, as previous studies have established a relationship between blink rate and the crow’s perceived sense of danger; specifically, blink rate is negatively correlated with activity in fear-associated brain regions, and crows decrease blink rate when faced with a threatening stimulus compared to while foraging (Marzluff et al., 2012; Cross et al., 2013). Although the image resolution was sufficient to see the white flash of the crow’s nictating membrane, the birds sometimes turned their heads such that their eyes were no longer visible, so we calculated an observed blink rate by dividing the number of observed blinks by the amount of time the eye was visible. The cage interior became too dark to observe blinks when the panels to the stimulus stage were closed, so we only calculated blink rate when the stimulus stage was visible during the seven reveals of the stimulus phase.

We measured the crows’ movement because physiological activity can confound the amount and location of FDG uptake within the brain (Bhargava et al., 2011). We quantified the following actions as 1 unit of movement: crow moved 5–50 cm laterally along the perch (did not count if it moved < 5 cm, counted as 2 units if moved > 50 cm), crow rotated its body 180° to face the opposite direction, and crow hopping from the perch to the cage floor (or vice-versa). Because we measured movement to account for possible confounds to FDG uptake activity, we counted movement throughout the entire 10 min of the stimulus phase, including when the stimulus stage interior was hidden from the crow’s view.

### Image Processing

After each crow was imaged, we conducted a 13 min attenuation scan, then reconstructed the image using the vendor-supplied 3D OSEM/MAP algorithm to an isotropic spatial resolution of 2.5 mm full width at half maximum, with attenuation and scatter corrections applied to the data. The image matrix was 128 × 128 × 159. We exported reconstructed images using DICOM for the statistical parametric analysis software.

We imported the raw DICOM data to ImageJ (Schneider et al., 2012), manually aligned their orientation to match the jungle crow (*Corvus macrorhynchos*) brain atlas established by Izawa and Watanabe (2007) and adapted for PET by Marzluff et al. (2012), and trimmed the images to include only the brain. We stereotactically aligned the scans by estimating and applying nine affine parameters to the images using algorithms originally designed for automated human brain analysis (NEUROSTAT, University of Utah; Minoshima et al., 1992), which have been adapted for crow brains analysis. We estimated alignment precision to be one-two pixels. Finally, we normalized all uptake values to a global brain FDG uptake.

Although we used Izawa and Watanabe’s (2007) atlas as a guide to identify the regions significantly activated by each stimulus, we did not use it as the sole determinant. This was because jungle crows are larger than American crows (Jungle crow mean male weight: 680 g, American crow: 450 g, Kitagawa, 1980; Kilham, 1990). More importantly, the atlas was based on a sectioned brain, whereas our activation foci were based on *in vivo* imaging. Sectioned brains tend to “flatten” (reduced *Y*-axis length, increased *X*- and *Z*- axis lengths) after being extracted from the skull and are vulnerable to other artifacts which can further alter the original shape (Rolls et al., 2008). Therefore, we scaled Izawa and Watanabe’s (2007) atlas for use with American crow brains and used it in conjunction with the shape and extent of the total activation (not just the focal coordinates) to determine the activated regions.

### Statistical Analyses

Due to the small sample size, we calculated differences between the first and second scan’s stimulus phase behaviors (blink rate, gaze, and movement) using a paired samples *t*-test, correlation between FDG uptake and blink rate/movement using a Pearson correlation test, and differences between the different stimuli of the 2nd scan using a linear model, all in RStudio version 1.0.136 (RStudio Team, 2016). We determined significant differences in regional activity within the brain using an automated voxel-wise subtraction and Z-statistic mapping algorithm originally designed for automated human paired-brain analysis (NEUROSTAT, University of Utah; Minoshima et al., 1992). This algorithm conducts a paired *Z*-test comparing the study population’s total difference in FDG signal strength between each individual subject’s first (control) and second (stimulus) scans against the study population’s pooled variance; it does this for each voxel coordinate throughout the entire brain. Due to the large number of comparisons made, the algorithm calculated a Z-threshold for statistical significance using a modified Bonferroni correction commonly utilized in imaging research (Friston et al., 1991). Because this threshold is conservative (Cross et al., 2013), we also report any regions with a Z-score more than 3.0, as these regions may be worth examining in greater detail in future studies. We verified the voxel-wise results by obtaining spherical volumes of interest (VOIs; 2-voxel radius) centered around the significant coordinates to determine if said results are driven by outlying individual scans.

### Ethical Note

We captured, housed, and tested all crows (including PET/CT scans) in accordance with the Institutional Animal Care and Use Committee of the University of Washington (IACUC; protocol number 3077-01), Federal Collecting Permit MB761139-0, and State of Washington Scientific Collection Permit 14-010. We released all crows back into the wild at the location where they were captured at the conclusion of the study.

## Results

We were only able to obtain usable imaging data from 13 of the 17 captured crows; the other four individuals had one of their scans invalidated by mechanical/software issues with the imaging system. Additionally, we were unable to obtain blink rate data from one bird’s 2nd scan (audio stimulus) due to it positioning itself with its eye remaining out of the camera’s field of view throughout the stimulus phase.

### Differential Brain Activity

Crows that were exposed to the unimodal visual stimulus showed no notable increases in brain activity relative to their initial baseline scan, even at the coordinates of peak differential activity for the combined stimulus (Supplementary Figure 2). See Supplementary Figure 3 for differential activity patterns throughout the entire brain in response to the visual stimulus.

**FIGURE 2:**
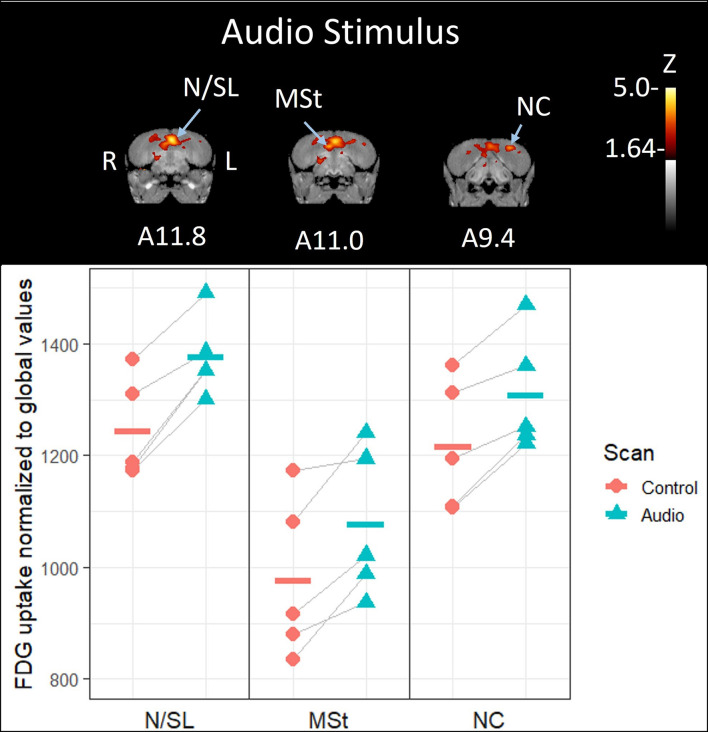
**Top:** Coronal view of voxel-wise subtractions (converted to Z-scores) showing differential brain activity at the indicated region for all crows exposed to the vocalizations of feeding crows during their stimulus scan (*n* = 5). Brain activity is superimposed atop a composite (*n* = 4) structural MRI of the American crow brain. Slice coordinates (A11.8, A11.0, and A9.4) refer to Izawa and Watanabe (2007) jungle crow atlas. **Bottom:** individual normalized (global) uptake values obtained from VOI’s centered on peak activation coordinates. Horizontal lines indicate group means. Note that only the nidopallium/lateral septum border (N/SL) showed significant increases in brain activity; the medial striatum (MSt) and caudal nidopallium (NC) did not meet the critical Z-threshold.

**FIGURE 3:**
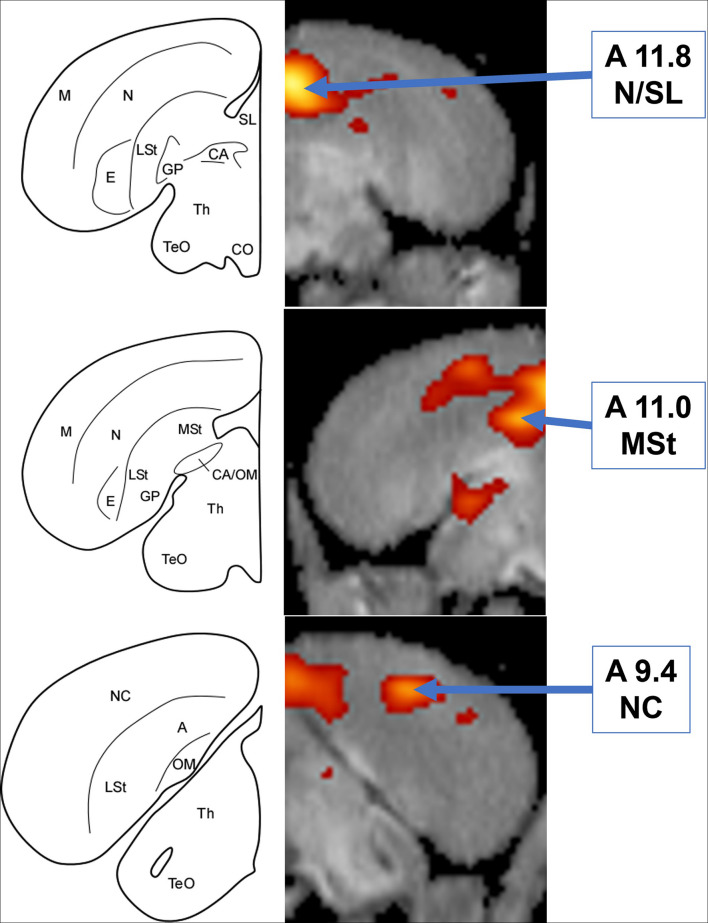
**Left:** Schematic coronal hemisections of the American crow brain, drawn based on structural MRI of the American crow brain and Izawa and Watanabe jungle crow atlas. A, Arcopallium; CA, Anterior Commissure; CO, Optic Chiasm; E, Entopallium; GP, Globus Pallidus; LSt, Lateral Striatum; M, Mesopallium; MSt, Medial Striatum; N, Nidopallium; NC, Caudal Nidopallium; OM, Occipito-mesencephalic Tract; SL, Lateral Septum; TeO, Optic Tectum; Th, Thalamus. **Right:** Coronal view of voxel-wise subtractions (converted to Z-scores) showing differential brain activity at the indicated region for all crows exposed to the audio stimulus (*n* = 5). Brain activity is superimposed atop a composite (*n* = 4) structural MRI of the American crow brain.

By contrast, crows that were exposed to the unimodal audio stimulus showed significantly increased activity in the medial portion of the nidopallium in their left hemisphere compared to their baseline control scan (10.6% increase, *Z* = 4.57, *P* < 0.001, Figures 2, 3); this region includes or is adjacent to Field L and possibly the lateral septum. This activity extends along the anterior-posterior axis with the most visible at A11.8. While no other regions’ increase in activity in response to the audio stimulus exceeded the critical Z-threshold (*Z* = 4.08), there was a notable increase in FDG uptake within a part of the medial striatum ventral to the highly activated medial nidopallium, particularly in the right hemisphere (10.2% increase, *Z* = 3.58, *P* < 0.001, at A11.0). We observed another low threshold (but notable) activity increase in the caudal nidopallium ventral to the HVC region in the left hemisphere (7.5% increase, *Z* = 3.33, *P* < 0.001, at A9.4). See Supplementary Figure 4 for differential activity patterns throughout the entire brain in response to the audio stimulus.

**FIGURE 4:**
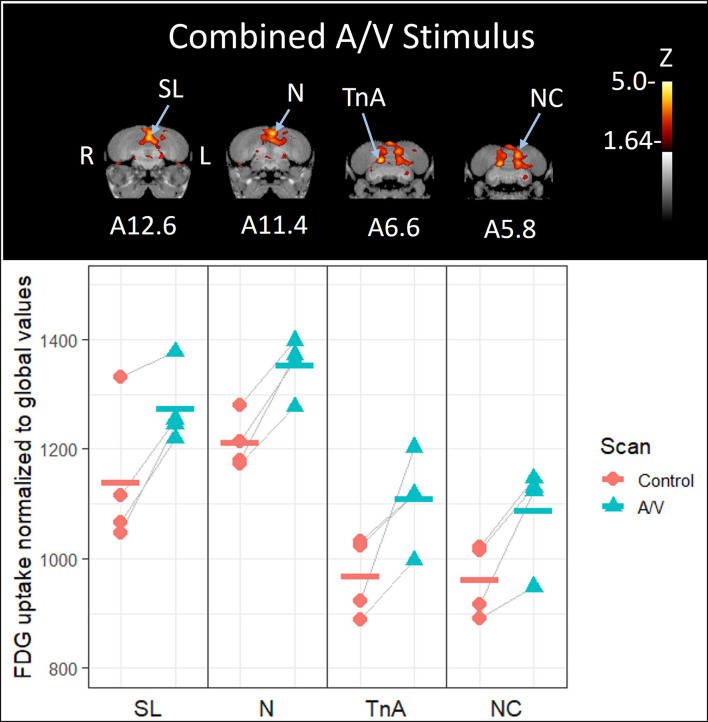
**Top:** Coronal view of voxel-wise subtractions (converted to Z-scores) showing differential brain activity at the indicated region for all crows simultaneously exposed to the vocalizations of feeding crows and the sight of their preferred food item during their stimulus scan (*n* = 4). Brain activity is superimposed atop a composite (*n* = 4) structural MRI of the American crow brain. Slice coordinates (A12.6, A11.4, A6.6, and A5.8) refer to the Izawa and Watanabe (2007) jungle crow atlas. **Bottom:** individual values for normalized (global) uptake obtained from VOI’s centered on peak activation coordinates. Horizontal lines indicate group means. Note that while the nucleus taeniae of the amygdala (TnA), and nidopallium (N) showed significant increases in FDG uptake, the lateral septum (SL) and caudal nidopallium (NC) did not meet the critical Z-threshold.

**FIGURE 5:**
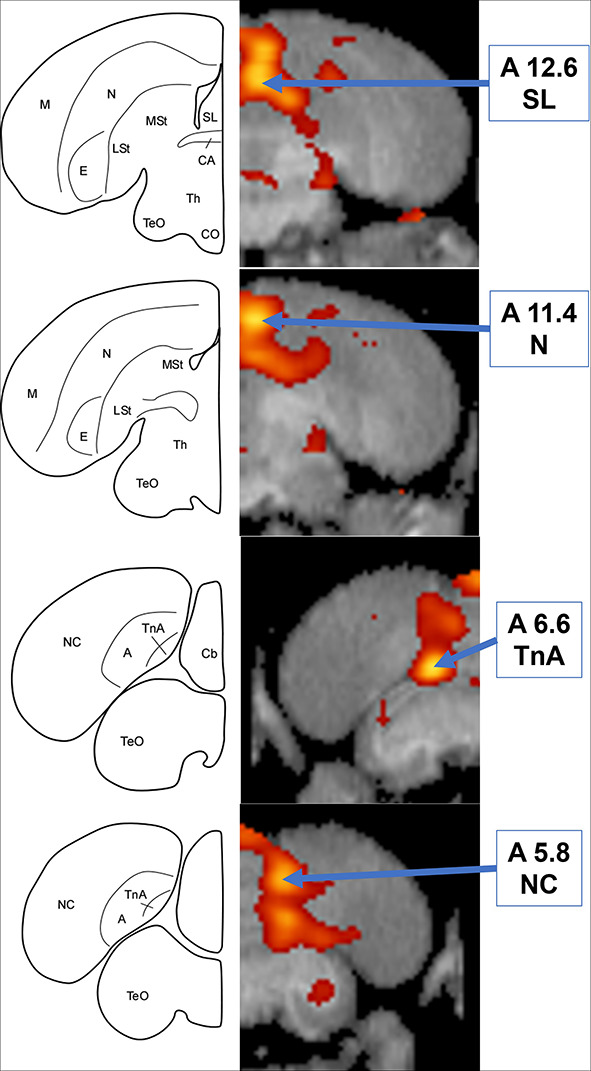
**Left:** Schematic coronal hemisections of the American crow brain, drawn based on structural MRI and Izawa & Watanabe jungle crow atlas. Cb, Cerebellum; TnA, nucleus taeniae of the amygdala. For other abbreviations, see Figure 3. **Right:** Coronal view of voxel-wise subtractions (converted to Z-scores) showing differential brain activity at the indicated region for all crows simultaneously exposed to the audio and visual stimuli (*n* = 4). Brain activity is superimposed atop a composite (*n* = 4) structural MRI of the American crow brain.

Simultaneous presentation of preferred food item and food-associated vocalizations induced higher FDG-uptake activity in those areas responding to audio stimuli in the left hemisphere (Figures 4, 5), including the medial nidopallium (11.6% increase, *Z* = 4.23, *P* < 0.001, at A11.4), lateral septum (11.8% increase, *Z* = 4.00, *P* < 0.001 at A12.6), and the caudal nidopallium (13.1% increase, *Z* = 3.66, *P* < 0.001, at A5.8) though the latter two were low threshold increases (Z-threshold = 4.14). We also observed significant increased activity in the TnA of the right hemisphere (14.7% increase, *Z* = 4.27, *P* < 0.001, at A6.6). See Supplementary Figure 5 for differential activity patterns throughout the entire brain in response to the combined A/V stimulus.

### Stimulus Phase Behavior

The crows visually attended to the stimulus stage whenever it was revealed, regardless of what was inside; they stared into the stage for a majority of the time (right eye: mean ± SD; 373.0 ± 48.0 s; left eye: 357.3 ± 87.3 s) that it was visible (420 s), with no significant difference in gaze time between their two scans (left eye: *t*_26_ = 1.51, *P* = 0.14; right eye: *t*_26_ = 0.56, *P* = 0.58) nor between any of the stimuli during their second scan [left eye: *F*_(2, 11)_ = 1.35, *P* = 0.30; right eye: *F*_(2, 11)_ = 0.31, *P* = 0.83]. Their mean blink rate remained steady (30.2 ± 8.3 blinks/min), with no significant change between the 1st and 2nd scans (*t*_11_ = 0.83, *P* = 0.43) nor any of the different stimuli presented during the 2nd scan [*F*_(2, 10)_ = 0.07, *P* = 0.93]. Most crows moved little during the stimulus phase, though there were several outlier individuals with a high degree of movement (3.4 ± 7.6 total movement, max = 39); there was no change in movement between the two scans (*t*_12_ = 1.23, *P* = 0.24) nor any of the different stimuli during the 2nd scan [*F*_(2, 11)_ = 2.12, *P* = 0.17]. See Supplementary Figure 6 for details on behavioral changes between scans and stimuli. We did not observe any significant correlation between blink rate or movement with the rate of FDG uptake (see Supplementary Figures 7, 8 for details).

## Discussion

When social animals gather around a food source, they must divide their attention between the activity of their fellow conspecifics and the food itself; the mental demand required to successfully navigate these situations likely necessitates increased neural activity in multiple regions and systems throughout the brain. Adult American crows have ample experience navigating the social milieu around a communal food source thus their relevant neural circuitry should be well-developed compared to other songbirds.

### Combined A/V and Emergent Activity

The combined A/V stimulus mimicked the conditions of a social feeding event; focal crows heard conspecifics vocalizing while simultaneously visually observing a preferred food item. These conditions triggered increased activity in two regions (the TnA and caudomedial nidopallium) that were not active in response to either unimodal stimulus, and further defined the activity of two regions (the lateral septum and medial nidopallium) that were active for the unimodal audio stimulus.

The largest increase in activity occurred in the TnA, a region associated with processing social information (Cheng et al., 1999; Mayer et al., 2019) and one of the regions we hypothesized would be active in response to hearing conspecific vocalizations. This suggests that the TnA is involved in integrating sensory information with social stimuli, which supports existing evidence linking the TnA with controlling social foraging behavior (Cheng et al., 1999, although see Xin et al., 2017). As the amygdala is also associated with processing a learned fear response (Cross et al., 2013), this result could alternatively be explained as the crows being especially frightened by some confounding factor associated with the combined A/V stimulus that was absent for the other stimuli. However, frightening stimuli usually decreases the blink rate of observing crows (Marzluff et al., 2012; Cross et al., 2013), which was not strong in our experiments (we observed only a slight reduction FDG uptake in the TnA; see Supplementary Figure 7). Finally, neither of the unimodal conditions prompted increased TnA activity. We therefore conclude that the TnA activity during the multimodal stimuli was not caused by a strong fear response.

The other region notably stimulated by the combined A/V stimulus, but not by either unimodal stimulus, was located medially within the caudal nidopallium. The caudomedial nidopallium is involved in avian auditory processing (Atoji and Wild, 2009; Moorman et al., 2012), and recent evidence suggests it may be a passerine-specific enlargement of the avian prefrontal area and involved with multimodal processing (von Eugen et al., 2020), which our results support.

The addition of the visual stimulus slightly enhanced the activity of the medial nidopallium and lateral septum; we observed their activity as a merged entity for the audio-only stimulus and separately as two distinct entities for the combined A/V stimulus. As in mammals, the avian lateral septum is a part of descending connections from the hippocampus to the brainstem limbic centers and is involved in regulating aggression (Cooper and Erickson, 1976; Goodson et al., 1998; Goodson, 2005). This is consistent with the fact that fights and status displays were common among crows jockeying for access to food when we recorded the stimulus audio, so there is a high likelihood that the audio stimuli contained aggressive vocalizations. The other region stimulated by both audio-associated conditions, the medial nidopallium, has been implicated with A/V stimulus processing in imprinting learning (Wallhäusser and Scheich, 1987; Bredenkötter and Braun, 1997), though those studies focused on precocial chicks of a species belonging to a different taxonomic order. Further studies are needed to determine whether the medial nidopallium in different avian species are equivalent in function.

While we observed activity in two *a priori* hypothesized regions associated with social behavior (the TnA and lateral septum), we did not observe any activity in the thalamus, the region we predicted would be active during the multimodal stimulus. The thalamus is involved with filtering and relaying sensory information to other brain regions (Bentivoglio et al., 1993); it’s possible that there was not enough contrast in sensory information presented between the control and stimulus scans. Although the audio stimulus increased the amplitude of the crow’s sensory environment, we kept the amount of light, non-stimulus ambient sound, and temperature constant for all scans, potentially masking any effect the added stimulus sounds might have had on thalamus activity.

### Response to Unimodal Audio Stimulus

In contrast to the previous regions, the medial striatum was only notably stimulated by the unimodal audio stimulus; this activity was not present when food visually accompanied the vocalizations. The avian medial striatum, extending in the antero-posterior axis, corresponds to the mammalian ventral striatum. In birds, the anterior portion is involved in sensory processing (e.g., area X in songbirds), though the activated area observed in the present study is in the limbic posterior region including the avian nucleus accumbens (Husband and Shimizu, 2011).

The other region notably activated by the audio-only condition is located centrally between the nidopallium and caudal nidopallium, ventral to the HVC; this area is most likely a lateral area of Field L. Like the caudomedial nidopallium, Field L is heavily involved in songbird auditory processing and filtering conspecific vocal signals from other sounds (Zaretsky, 1978; Wild et al., 1993; Grace et al., 2003; Nagel et al., 2011). Despite its central role in the avian auditory network, Field L was not stimulated when the listening crows could also see food, which further emphasizes the importance of context in songbird auditory processing of a vocal signal.

### Lateralization of Activity

Avian brains are highly lateralized (Mench and Andrew, 1986), and we observed some bias in hemispherical activity in all observed regions. Most of the strongest observed activity occurred in the left hemisphere, though the TnA and medial striatum were most active in the right hemisphere. As there was no significant difference in gaze direction (see Supplementary Figure 6), this asymmetry cannot be explained because of directional sensory bias. Previous studies have found that the caudomedial nidopallium tends to be more active in the left hemisphere for passerine songbirds when processing audio information (Moorman et al., 2012; Ocklenburg et al., 2013), which is consistent with our findings.

### Lack of Response to Unimodal Visual Stimulus—Why More Activity for Vocalizations?

In contrast to the multiple active regions that we observed in response to conspecific vocalizations, we did not observe any areas of increased activity (*a priori* hypothesized hypothalamus or otherwise) in response to the unimodal visual stimulus of a preferred food item. This lack of activity was not the result of a single outlier individual’s contrary activity masking a majority trend (Supplementary Figure 4). While it’s possible that some unknown factor was occurring to reduce food-associated brain activity (such as imaging-associated stress inhibiting appetite) or the metabolic uptake of the radiotracer (such as increasing blood flow to the GI tract in anticipation of a meal), we believe this is unlikely for the simple reason that the food caused observable changes in brain activity when added to the auditory-associated stimuli.

Why did the food, something necessary for the crow’s survival, evoke less activity throughout the brain than the vocalizations? We posit that the food was less interesting to the crows and required less cognitive power to neuronally process than the calls. The food did not add light to the crows’ sensory environment (only a negligible increase in visual signals sent to the brain), remained static (less cognitive processing required), and, because they were fed after their previous control scan, was something they had already experienced in the context of the imaging lab (less stimulating). By contrast, the calls added sound to the crows’ sensory environment (large increase in auditory signals sent to the brain), were dynamic (more cognitive processing required), and were likely much more surprising to hear within the setting of the imaging lab (more stimulating). When a more interesting stimulus (the vocalizations) was present to maintain the crows’ attention, this may have had the secondary effect of encouraging the crows to pay more attention by proxy to the food than they would otherwise.

The social aspect of the vocalizations is another factor to consider. Although we recorded the stimulus vocalizations from crows as they gathered around a food source, crows in such contexts usually do not limit their communication to food-associated information; for example, they also vocalize to announce their presence, assert dominance, recruit allies, etc. (Pendergraft and Marzluff, 2019). In addition to the overt signals, their vocalizations also contained characteristics that can be used to identify the caller’s sex and identity (Mates et al., 2015). The amount of social information contained within the vocalizations, combined with the information being conveyed indirectly (they contained information *about* things, whereas the food *was* food) is a likely reason why the calls elicited significant increases in neural activity throughout the brain, yet the food did not.

The amount of neuronal processing required to extract all the social information from a vocal signal is supported by behavioral observations, as most birds closely attend to conspecific vocalizations (Dooling and Prior, 2017). Pinyon jays (*Gymnorhinus cyanocephalus*), another Corvidae species, pay most attention to conspecific vocalizations when they recognize the caller as belonging to their own flock (Marzluff and Balda, 2010), which may be applicable here: there’s evidence that American crows can use acoustic properties of the vocalization to identify the caller (Mates et al., 2015) and we observed increased activity in the caudomedial nidopallium- a region associated with vocal recognition of known individuals (Chew et al., 1996; Bolhuis and Gahr, 2006). As we had pre-baited the capture site each day of the week preceding their capture and many of the crows had fallen into the habit of visiting the location each morning, it’s possible that the experimental crows may have recognized some of the callers from the stimulus tracks, which would further motivate them to attend the audio stimulus.

### No Confounding Effects of Repeated Testing

In a prior study where we balanced the presentation of four stimuli to crows, we observed that after crows experienced a potentially dangerous stimulus in one trial, their subsequent responses were biased toward fearful reactions (Swift et al., 2020). We did not observe such carryover effects in the present study; neither blink rate nor movement varied from the first to the second scan. This is likely due to the benign, non-threatening stimulus presented during the control scan as well as the limited number of presentations (two) each bird received.

## Conclusion

Taken as a whole, the six distinct regions activated by the sound of conspecifics vocalizing at a food source are associated with either processing auditory sensory data or social information. This would suggest that all the identified regions are either involved in a larger socio-auditory processing system or, more likely, are components of two or more brain systems that are triggered when the bird needs to process additional modalities or contextual information. Although multiple studies have examined avian brain activity in response to social interactions, this study is the first to use functional imaging to holistically measure activity in response to social signals under the context of communal foraging, one of many social contexts that birds regularly encounter in nature. The visual presence of food, despite not causing any notable changes in activity on its own, significantly alters the neural activity triggered by the sound of conspecifics vocalizing and even stimulates activity in regions not activated by either modality alone. This demonstrates that the context associated with a stimulus matters to the neuronal processing of that information, especially for something as varied as social interaction.

## Data Availability Statement

The raw data supporting the conclusions of this article will be made available by the authors, without undue reservation.

## Ethics Statement

The animal study was reviewed and approved by the Institutional Animal Care and Use Committee of the University of Washington (IACUC; protocol number 3077-01).

## Author Contributions

LP: conceptualization, funding acquisition, animal care, investigation, data curation and analysis, and writing—original draft. JM: conceptualization, investigation, funding acquisition, project administration, supervision, and writing—review and editing. DC: methodology, supervision, data curation and analysis, and writing—review and editing. TS: methodology, validation, and writing—review and editing. CT: methodology, funding acquisition, and writing—review and editing. All authors contributed to the article and approved the submitted version.

## Conflict of Interest

The authors declare that the research was conducted in the absence of any commercial or financial relationships that could be construed as a potential conflict of interest.

## Publisher’s Note

All claims expressed in this article are solely those of the authors and do not necessarily represent those of their affiliated organizations, or those of the publisher, the editors and the reviewers. Any product that may be evaluated in this article, or claim that may be made by its manufacturer, is not guaranteed or endorsed by the publisher.
