# Supplementary material for: American Crow Brain Activity in Response to Conspecific Vocalizations Changes When Food Is Present
Source: Front Physiol. 2021 Nov 18;12:766345. doi: 10.3389/fphys.2021.766345 (PMC8637333; doi:10.3389/fphys.2021.766345)
Supplement: Supplementary file 1 [file Data_Sheet_1.docx]

**Supplementary Material**

| Name | Action | Time (mm:ss) |
| --- | --- | --- |
| Injection | Interperitoneal FDG injection | 00:00 |
| Pre-stimulus phase | Crow returned to covered cage, stimulus stage is moved into position | 00:00 – 03:00 |
| Stimulus phase | Open panels, revealing stimulus stage interior | 03:00 |
|  | Close panels, hiding stimulus stage interior | 04:00 |
|  | Open panels, revealing stimulus stage interior | 04:30 |
|  | Close panels, hiding stimulus stage interior | 05:30 |
|  | Open panels, revealing stimulus stage interior | 06:00 |
|  | Close panels, hiding stimulus stage interior | 07:00 |
|  | Open panels, revealing stimulus stage interior | 07:30 |
|  | Close panels, hiding stimulus stage interior | 08:30 |
|  | Open panels, revealing stimulus stage interior | 09:00 |
|  | Close panels, hiding stimulus stage interior | 10:00 |
|  | Open panels, revealing stimulus stage interior | 10:30 |
|  | Close panels, hiding stimulus stage interior | 11:30 |
|  | Open panels, revealing stimulus stage interior | 12:00 |
|  | Close panels, hiding stimulus stage interior | 13:00 |
| Pre-imaging phase | Crow removed from cage and induced with isoflurane. Anesthetized crow secured to scanner bed and positioned within imager field of view | 13:00 – 26:00 |
| Imaging phase | Begin micro-PET scan | 26:00 |

*Table S1. Timeline of the experimental methodology between FDG injection and start of imaging process.*


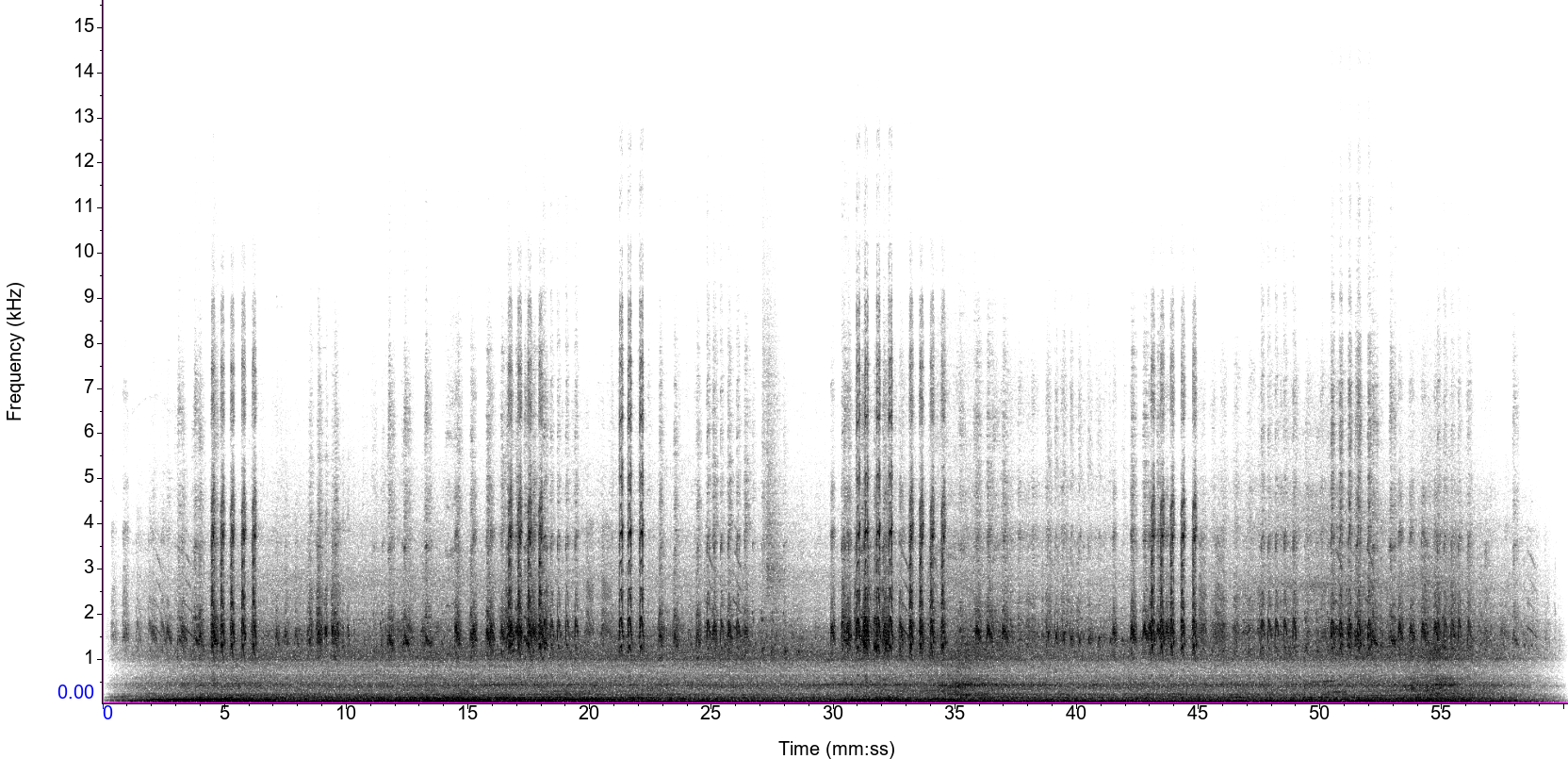


*Figure S1. Spectrogram of audio stimulus exemplar.*


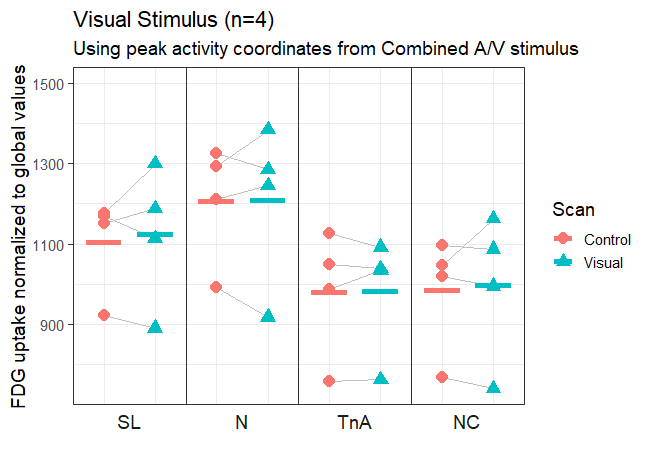


*Figure S2. Individual values for normalized (global) uptake of the birds exposed to the visual-only stimulus obtained from VOI’s centered on peak activation coordinates from the Combined A/V stimulus. Horizontal lines indicate group means.*


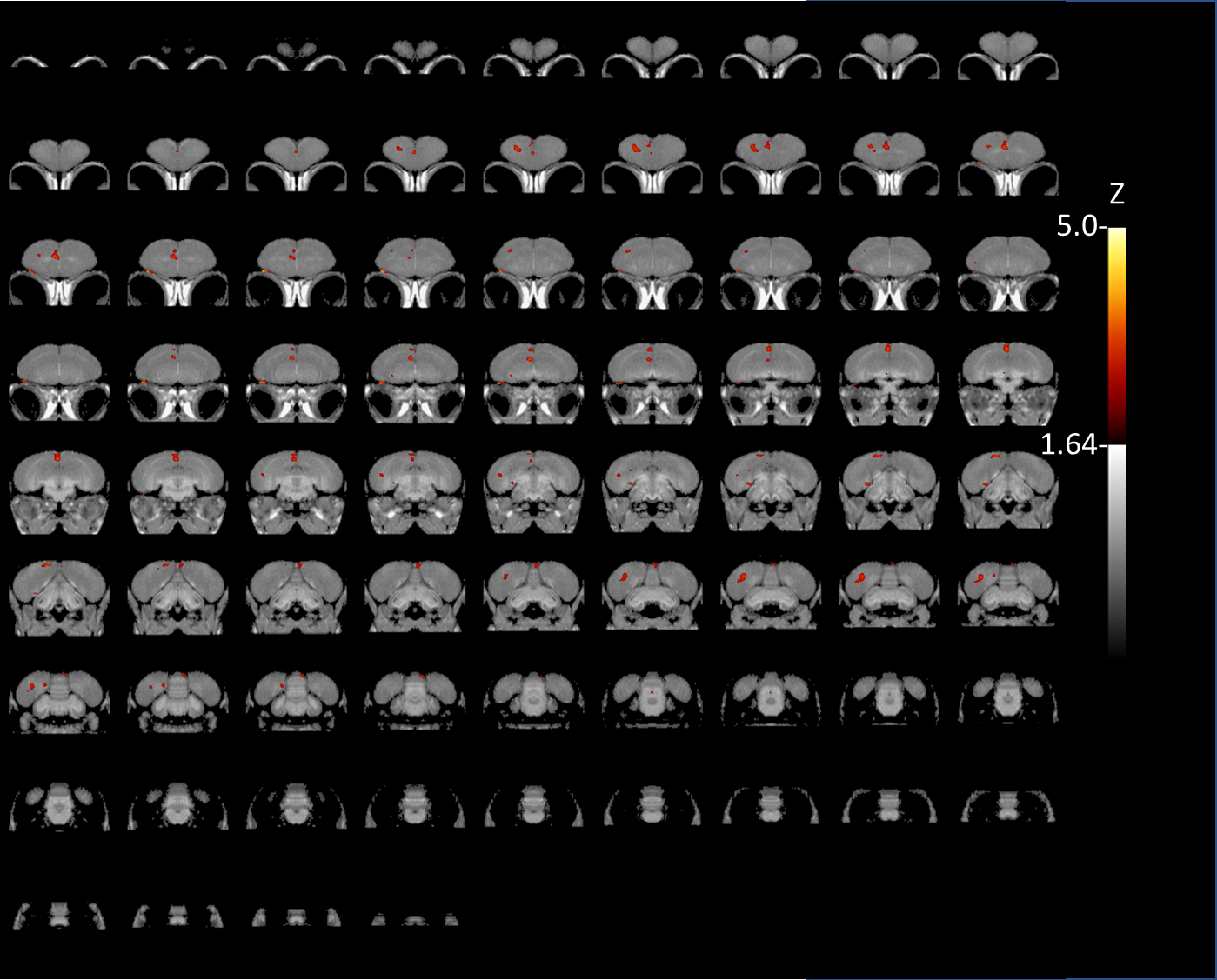


*Figure S3. Voxel-wise subtractions (converted to Z-scores) showing differential activity patterns throughout the brain for all crows (n=4) exposed to the sight of their preferred food item during their stimulus scan. Brain activity is superimposed atop a composite (n=4) structural MRI of the American crow brain.*


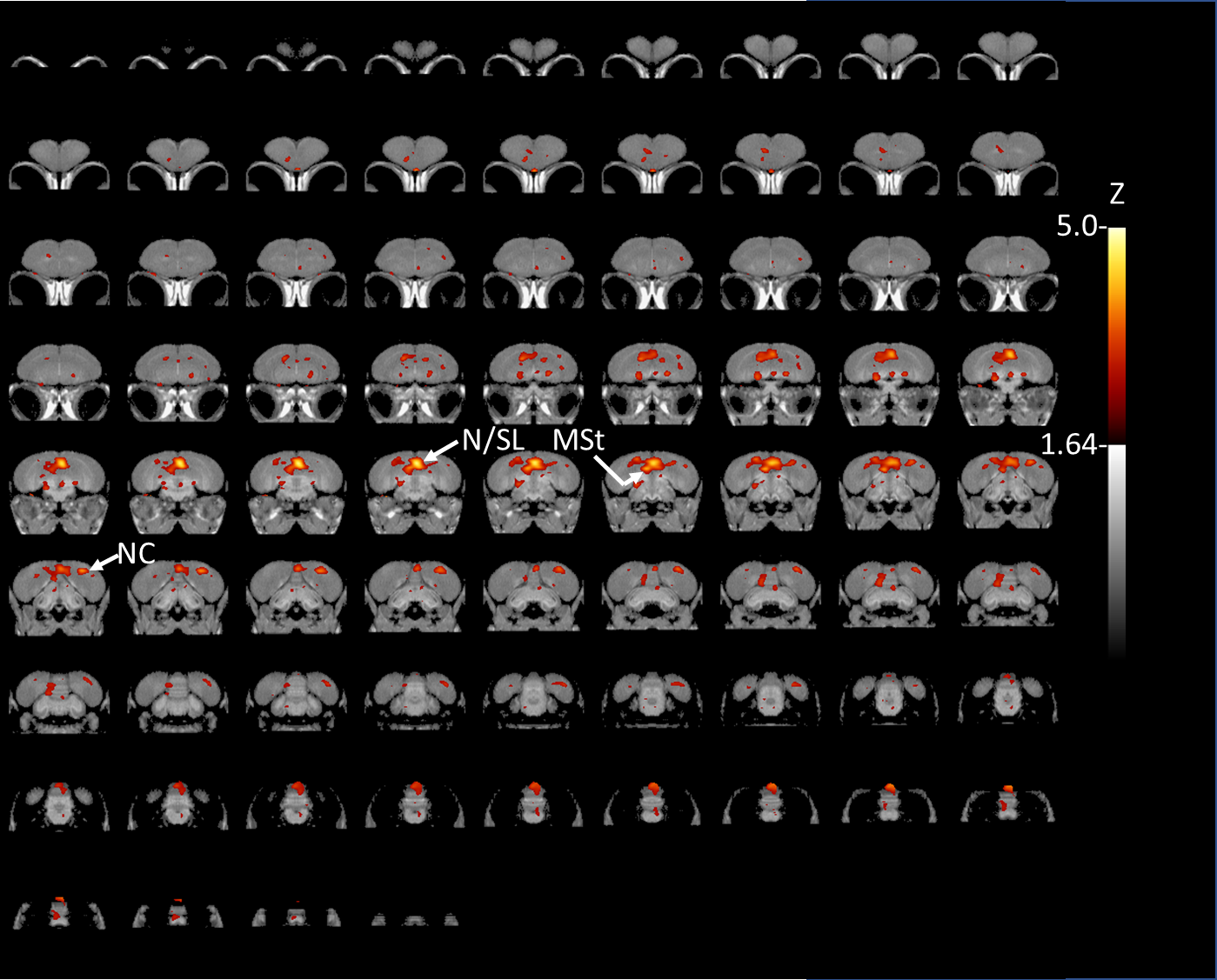


*Figure S4. Voxel-wise subtractions (converted to Z-scores) showing differential activity patterns throughout the brain for all crows (n=5) exposed to the vocalizations of feeding crows during their stimulus scan. Brain activity is superimposed atop a composite (n=4) structural MRI of the American crow brain. Note that only the nidopallium/lateral septum border (N/SL) showed significant increases in brain activity; the medial striatum (MSt) and caudal nidopallium (NC) did not meet the critical Z-threshold.*

*
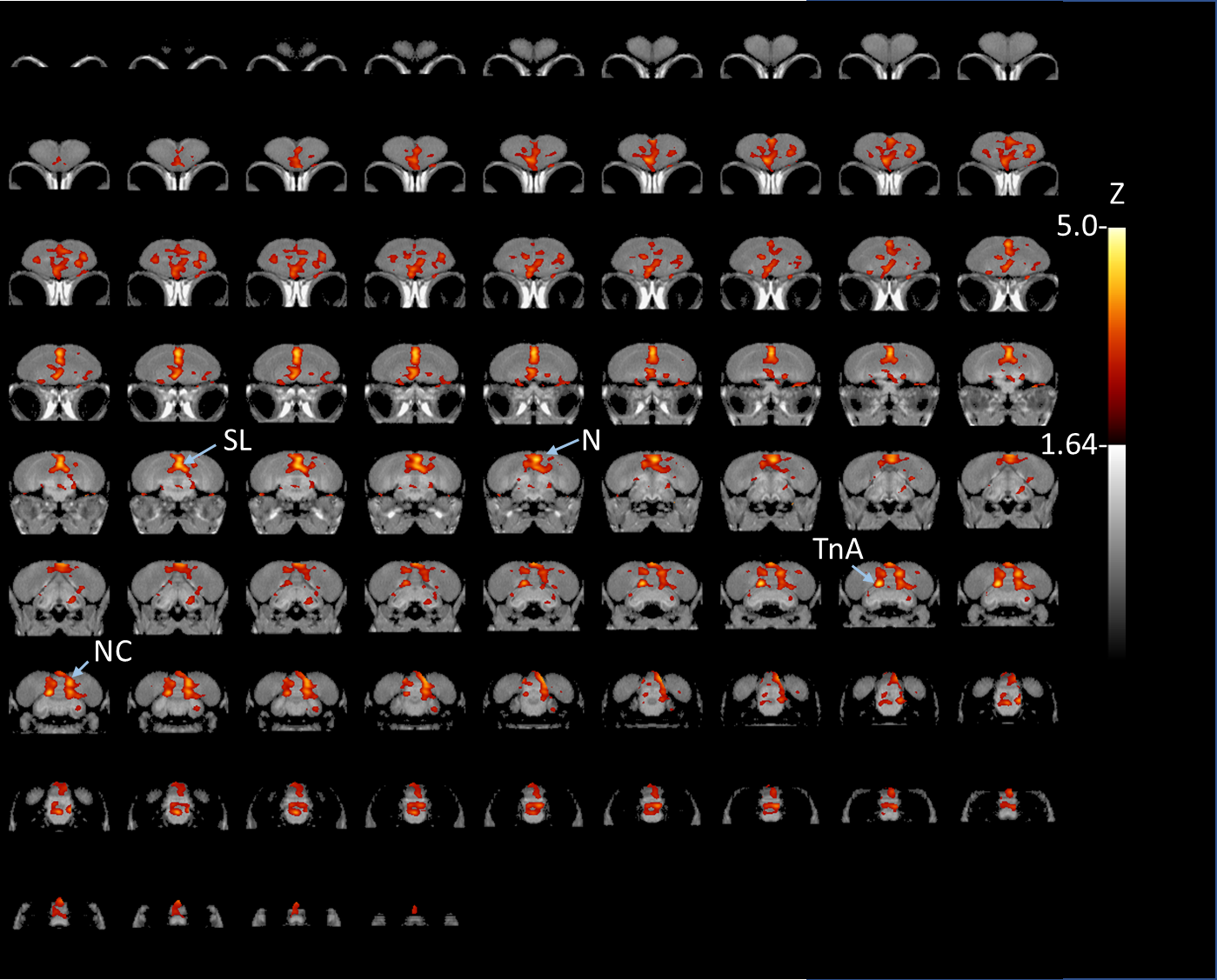
*

*Figure S5. Voxel-wise subtractions (converted to Z-scores) showing differential activity patterns throughout the brain for all crows (n=4) simultaneously exposed to the vocalizations of feeding crows and the sight of their preferred food item during their stimulus scan. Brain activity is superimposed atop a composite (n=4) structural MRI of the American crow brain. Note that while the nucleus taeniae of the amygdala (TnA), and nidopallium (N) showed significant increases in FDG uptake, the lateral septum (SL) and caudal nidopallium (NC) did not meet the critical Z-threshold.*


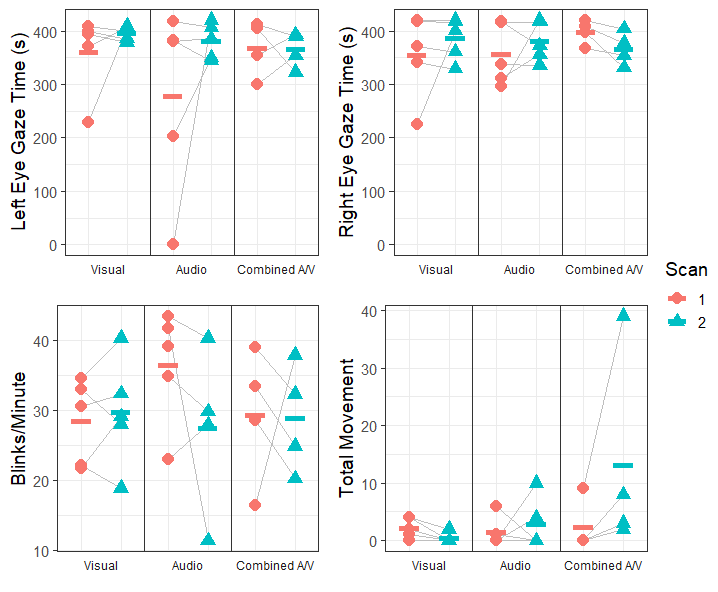


*Figure S6. Changes in behavior between the first and second scan of each individual crow for blink rate (top left), total movement (top right), and gaze time (left eye: bottom left; right eye: bottom right). Horizontal lines indicate group mean.*

*
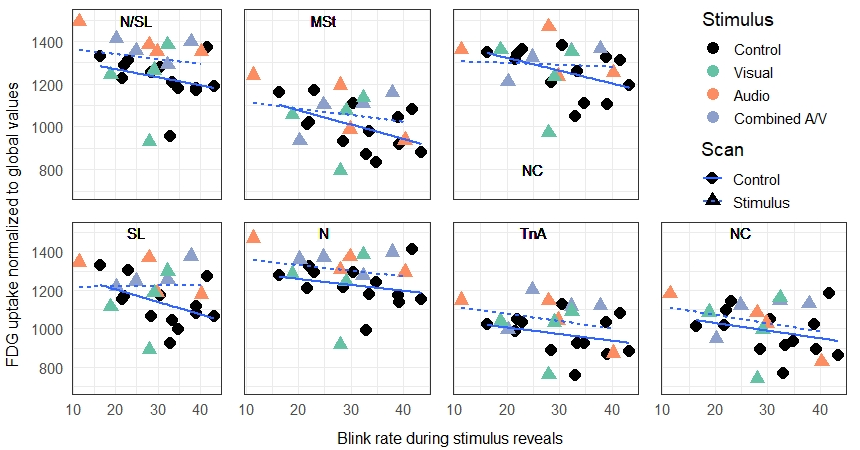
*

*Figure S7. Results of correlational analyses between blink rate and FDG uptake. Blink rate was not significantly correlated with FDG uptake in any region, for either the first control scan or second stimulus scan.* ***Top row****: regions significantly activated by the audio stimulus; N/SL (control scan: r= -0.31, t_11_= -1.09, P= 0.31; stimulus scan: r= -0.13, t_10_= -0.42, P= 0.68), MSt (control scan: r= -0.51, t_11_= -0.98, P= 0.07; stimulus scan: r= -0.20, t_10_= -0.65, P= 0.53), and NC (control scan: r= -0.46, t_11_= -1.73, P= 0.11; stimulus scan: r= -0.06, t_10_= -0.18, P= 0.86).* ***Bottom row****: regions significantly activated by the combined A/V stimulus; SL (control scan: r= -0.46, t_11_= -1.71, P= 0.11; stimulus scan: r= 0.02, t_10_= 0.06, P= 0.95), N (control scan: r= -0.25, t_11_= -0.86, P= 0.41; stimulus scan: r= -0.17, t_10_= -0.56, P= 0.59) TnA (control scan: r= -0.28, t_11_= -0.98, P= 0.35; stimulus scan: r= -0.24, t_10_= -0.79, P= 0.45), and NC (control scan: r= -0.28, t_11_= -0.98, P= 0.35; stimulus scan: r= -0.25, t_10_= -0.83, P= 0.42).*


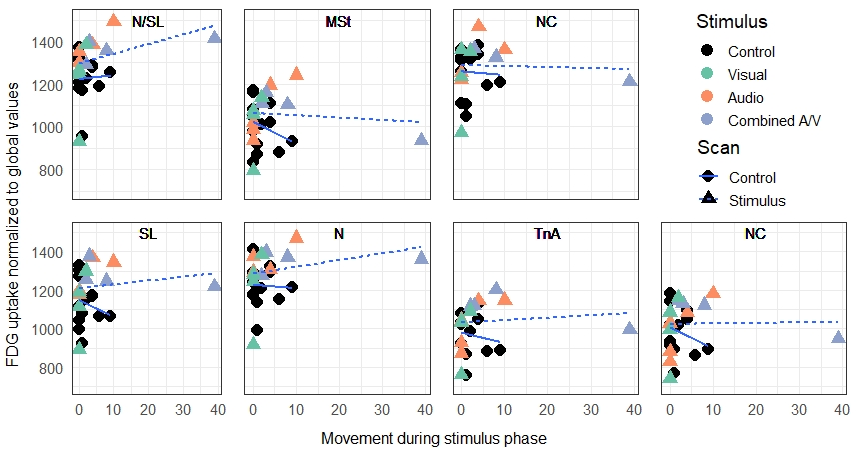


*Figure S8. Results of correlational analyses between movement and FDG uptake. Movement was not significantly correlated with FDG uptake in any region, for either the first control scan or second stimulus scan.* ***Top row****: regions significantly activated by the audio stimulus; N/SL (control scan: r= 0.05, t_11_= 0.17, P= 0.87; stimulus scan: r= 0.36, t_11_= 1.27, P= 0.23), MSt (control scan: r= -0.26, t_11_= -0.91, P= 0.38; stimulus scan: r= -0.09, t_11_= -0.32, P= 0.76), and NC (control scan: r= -0.05, t_11_= -0.15, P= 0.88; stimulus scan: r= -0.04, t_11_= -0.14, P= 0.89).* ***Bottom row****: regions significantly activated by the combined A/V stimulus; SL (control scan: r= -0.21, t_11_= -0.71, P= 0.49; stimulus scan: r= 0.16, t_11_= 0.55, P= 0.59), N (control scan: r= -0.05, t_11_= -0.16, P= 0.87; stimulus scan: r= 0.28, t_11_= 0.97, P= 0.35), TnA (control scan: r= -0.15, t_11_= -0.50, P= 0.63; stimulus scan: r= 0.11, t_11_= 0.36, P= 0.73), and NC (control scan: r= -0.27, t_11_= -0.91, P= 0.38; stimulus scan: r= 0.02, t_11_= 0.07, P= 0.94).*
